# Comparison of GentleWave system and passive ultrasonic irrigation with minimally invasive and conventional instrumentation against LPS in infected root canals

**DOI:** 10.1038/s41598-022-08835-4

**Published:** 2022-03-22

**Authors:** Johnathan P. Velardi, Theeb A. Alquria, Rayyan A. Alfirdous, Bruna J. M. Corazza, Ana P. M. Gomes, Eduardo G. Silva, Ina L. Griffin, Patricia A. Tordik, Frederico C. Martinho

**Affiliations:** 1 Division of Endodontics, Department of Advanced Oral Sciences and Therapeutics, University of Maryland, School of Dentistry, Baltimore, MD USA; 2 Department of Restorative Dentistry, Endodontic Division, São Paulo State University (Unesp), Institute of Science and Technology, São José dos Campos, Brazil; 3 Department of Social and Pediatric Dentistry, Institute of Science and Technology, São Paulo State University (Unesp), Institute of Science and Technology, São José dos Campos, Brazil

**Keywords:** Root canal treatment, Dentistry, Endodontics

## Abstract

This study compared the effectiveness of GentleWave system (GWS) and passive ultrasonic irrigation (PUI) in removing lipopolysaccharides (LPS) from infected root canals after minimally invasive (MIT) and conventional instrumentation (CIT) techniques. Sixty first premolars with two roots were inoculated with fluorescent LPS conjugate (Alexa Fluor 594). Of those, twelve were dentin pretreated, inoculated with fluorescent LPS conjugate, and submitted to confocal laser scanning microscopy (CLSM) to validate the LPS-infection model. Forty-eight teeth were randomly divided into treatment groups: GWS + MIT, GWS + CIT, PUI + MIT, and PUI + CIT (all, n = 12). Teeth were instrumented with Vortex Blue rotary file size 15/0.04 for MIT and 35/0.04 for CIT. Samples were collected before (s1) and after a root canal procedure (s2) and after cryogenically ground the teeth (s3) for intraradicular LPS analysis. LPS were quantified with LAL assay (KQCL test). GWS + MIT and GWS + CIT were the most effective protocols against LPS, with no difference between them (p > 0.05). PUI + CIT was more effective than PUI + MIT (p < 0.05) but less effective than GWS + MIT and GWS + CIT. GWS was the most effective protocol against LPS in infected root canals using MIT and CIT techniques.

## Introduction

Primary endodontic infection is a polymicrobial infection comprised of gram-negative bacteria species^1–3^. Lipopolysaccharides (LPS) are the main virulent factor present in the outer cell wall of gram-negative bacteria^4^. High LPS levels in root canal infection are associated with the development of symptoms of endodontic origin such as spontaneous pain, pain on palpation, tenderness to percussion^5–7^ and severity of periapical bone destruction^7,8^.

Several disinfection protocols have been tested against LPS in root canal infections over the years^9–13^. Previous in vitro and in vivo studies evaluated the ability of different instrumentation techniques using single file or multiple files systems and irrigation protocols to eliminate LPS^9–12^. Given their limited effectiveness in eradicating LPS from root canals, supplemental treatments with passive ultrasonic irrigation (PUI) and photodynamic therapy (PDT) have been investigated^13–15^. However, there is still room for improvement in eradicating LPS from root canals.

The innovative GentleWave System (GWS) (Sonendo, Inc, Laguna Hills, CA) is designed to promote optimal debridement, cleaning, and disinfection of the root canal systems with minimal instrumentation^16^. This advancement in technology has been explored recently^16–25^. The GWS is a multi-sonic ultra cleaning technology that uses advanced fluid dynamics, acoustic energy, and tissue dissolution chemistry. The manufacturer claims that the high-tech irrigation process of the GWS allows fluid to penetrate and clean complex root canal anatomy, optimizing root canal disinfection. Previous studies demonstrated promising results with GWS in cleaning and removing biofilms^17,19^, intracanal bacterial DNA^19^, calcium hydroxide medication^16–22^, residual debris^23,24^, separated instruments^25^, gutta-percha/sealer in retreatment^18,21^ and calcifications^20^. Until now, there have been no studies evaluating the effectiveness of the GWS against LPS in infected root canals.

In this study, we evaluated the effectiveness of the GentleWave system (GWS) in removing LPS from infected root canals after minimally invasive (MIT) and conventional instrumentation (CIT) techniques. Second, we investigated the ability of passive ultrasonic irrigation (PUI) in disinfecting LPS after MIT and CIT. Lastly, we compared GWS versus PUI in LPS disinfection. The null hypothesis tested here was no difference between GWS and PUI in eliminating endotoxins from minimally or conventionally prepared infected root canals.

## Results

No LPS was detected in the negative control after gamut radiation with cobalt 60 used to degrade preexisting LPS. We recovered no LPS from swab samples collected from the external surface of the root prior to grinding. LPS was detected in all twelve samples analyzed under CLSM validating the fluorescent LPS-dentin infection model. The mean LPS depth penetration was 400 µm into dentin; Table 1 shows the amount of LPS encountered in all groups at different sampling times (s1-s3). At s1, LPS was recovered from all root canal samples (48/48). GWS + MIT and GWS + CIT were the most effective protocols against LPS, with no difference between them (p > 0.05) (Table 1). PUI + MIT had the smallest effect. PUI + CIT was more effective than PUI + MIT (p < 0.05) but less effective than GWS + MIT and GWS + CIT (Table 1). LPS was recovered in 100% of the root canals after PUI + MIT and PUI + CIT at s2 and s3. In contrast, no LPS was detected in 42% of the root canals after GWS + MIT and 50% of the root canals after GWS + MIT at s2 and s3.

**Table 1:** Lipopolysaccharides (LPS) content (mean ± SD) found in each group at different sampling times (s1, s2, and s3).

Sample time	SI (before root canal procedure)	s2 (after root canal procedure)	s3 (after cryogenic grind)
Area sampled	Lumen of the canal	Lumen of the canal	Intraradicular
Groups/sample technique	Paper point sample	Paper point sample	Cryogenic ground
MIT + PUI	9.84 ± 0.70 EU/mL Aa	4.05 ± 1.24 EU/mL Ba	7.19 ± 0.33 EU/mL ABa
CIT + PUI	9.91 ± 0.46 EU/mL Aa	1.85 ± 0.70 EU/mL Bb	1.72 ± 0.26 EU/mL Bb
MIT + GWS	9.79 ± 0.95 EU/mL Aa	0.43 ± 0.11 EU/mL Bc	0.55 ± 0.14 EU/mL Bc
CIT + GWS	9.82 ± 0.68 EU/mL Aa	0.38 ± 0.27 EU/mL Bc	0.41 ± 0.19 EU/mL Bc

Different uppercase letters mean intragroup significant difference (p < 0.05).

Different lowercase letters = intergroup significant difference (p < 0.05).

*MIT* Minimally Invasive Instrumentation Technique, *CIT* Conventional Instrumentation Technique, *PUI* Passive Ultrasonic Irrigation, *GWS* GentleWave System.

## Discussion

Minimal instrumentation has been attracting interest among clinicians and researchers lately^26,27^. Special attention has been given to root dentin preservation by instrumenting the canals with smaller instrument sizes than conventionally used. However, a special concern raised is whether the root canal enlargement achieved with minimal instrumentation is enough to properly promote the root canal system's disinfection. In this study, we successfully compared the effectiveness of GWS and PUI in disinfecting LPS from the root canal system after minimally invasive (MIT) and conventional instrumentation (CIT).

This study devised a novel LPS dentin infection model using LPS labeled with Alexa Fluor 594 dye. This Alexa Fluor 594 dye is a bright, red-fluorescent dye that can be excited at 590/617 and visualized under confocal laser scanning microscopy (CLSM). Previous studies utilized LPS fluorescently labeled to follow LPS binding, transport, and cell internalization^28,29^. However, this is one of the first study to explore LPS fluorescently labeled in root canals. To validate this novel LPS-infection model, twelve teeth were pretreated to open the dentinal tubules, inoculated with LPS labeled with Alexa Fluor 594 dye, and then submitted to CLSM. Here, we detected fluorescent LPS in 100% of the CLSM’s samples. The fluorescent LPS allowed us to track the LPS depth penetration into dentin using the CLSM. The CLSM analysis indicated a mean LPS depth penetration of 400 into the dentin. Such a finding is consistent with Horiba et al., which showed significant LPS content in 300 µm depth, ranging from 100 to 800 µm, in teeth with the radiolucent area^8^. One profound advantage of the new LPS-dentin-infection model is that the LPS used for inoculation is already conjugated with the dye, and therefore it is not required to stain the dentin substrate, which eliminates possible background false-positive signals from the stained substrate.

Unlike previous LPS studies that sampled root canals solely with paper points (PP)^5–7,10–15^, we cryogenically ground/pulverized the teeth for intraradicular LPS analysis. This is an innovative technique previously unexplored. Although the PP sampling technique is commonly used to sample LPS from root canal infections, it has several limitations. Among them, the PP is restricted to the main canal, not sampling complex areas of the root canal system such as isthmuses, ramifications, and dentinal tubules, which may harbor LPS, therefore possibly underestimating the total amount of LPS present in an infection. Previous studies have successfully used similar grinding protocols to explore bacterial analyses^1,30^. In this study, twelve additional teeth with no LPS inoculation and no treatment were submitted to gamut radiation with cobalt 60 (20 KGy for 6 h) as a negative control for the preexisting LPS degradation protocol. The ability of gamut radiation with cobalt 60 to degrade preexisting endotoxin is described elsewhere^31^. Currently, no LPS was detected in all negative controls for the gamut radiation. Moreover, all the swab samples collected from the external surface of the root and crown before grinding showed no LPS with the KQCL test.

Here, we compared the GWS and PUI in LPS disinfection. While ultrasonic irrigation depends on the acoustic energy from an oscillating file in which the file motion is likely limited in narrow canals^32^, the GW system uses a broad spectrum of sound waves to distribute fluids throughout the root canal. In contrast to ultrasonic energy dispersed at a single frequency in the PUI, the multisonic energy emitted by the GWS enables effective delivery of energized irrigation into microsized dentinal tubules at high flow^33^.

Our results showed that GWS + MIT and GWS + CIT were the most effective disinfection protocols. Here, we performed the MIT with ISO file size #15. An important advantage of the GWS is that the treatment tip is placed in the pulp chamber and not inside the canals allowing a minimally invasive root canal instrumentation. Although the manufacturer suggests no need for root canal enlargement beyond ISO size 15, we included one experimental group enlarged to ISO size 35/0.04 (GWS + CIT-group) to allow a more standardized comparison to the PUI + CIT.

PUI + MIT showed the smallest effect against LPS as expected. This result may be attributed to the limited root canal enlargement with an ISO file size #15. Unlike the GWS, to achieve optimal disinfection with PUI, the file or ultrasonic tip needs to be taken to the apical third of the root canal and move freely inside, avoiding touching the walls; hence the canals need a significantly greater enlargement than the ISO size 15 performed in this group. Previous studies have demonstrated that the direct physical contact of the PUI file with the canal wall can affect its motion and result in less acoustic microstreaming and, in turn, less cleaning effectiveness^34^. Moreover, the contact of the PUI file to the canal walls can restrict the transverse displacement amplitude of the file, substantially reducing the acoustic microstreaming^34^. It is worth pointing out that the 30G needle could not be introduced to 2 mm short of the WL in the minimally instrumented canal to ISO file size #15, which may have contributed to the small effect of PUI + MIT against LPS found here. Previous investigations showed that a minimum size and preparation are needed for adequate irrigation^35^.

PUI + CIT significantly reduced LPS from infected root canals, but it was less effective than GWS + MIT and GWS + CIT. The effectiveness of PUI in reducing LPS found here is consistent with previous investigations^14,15,36^. However, variations across the study’s protocol limits comparisons. Here, we activated NaOCl with a SATELEC sonofile (ACTEON Inc. Mount Laurel, NJ, USA) K-file ultrasonic tip size 15 for 1 min in each 2 mm shorter of the root canal length at a power setting of 4 using a ProUltra Piezo Ultrasonic unit (Dentsply Sirona, Charlotte, NC, USA). Herrera et al. flooded the root canal with 17% ETA and activated it for 30 s using an ultrasonic tip inserted 2 mm shorter of the root canal length with a power setting of 30%^36^. Aveiro et al. activated 6% NaOCl with three cycles of 20 s operating at 10% with the ultrasonic insert tip positioned 2 mm short of the WL^14^. While Hasna et al. investigated the effectiveness of PUI against LPS using 2.5% NaOCl and 3% NaOCl gel using an ultrasonic tip at WL with 10% frequency^15^. Despite clinical protocol differences, all these previous investigations^14,15,36^ show better LPS removal with PUI than without.

GWS + MIT and GWS + CIT were the most effective protocols against LPS. Foremost, this study showed the high effectiveness of the GWS in reducing LPS from the main canal as indicated by the PP sampling technique and intraradicular as revealed by the cryogenic grinding samples. While LPS was detected in all samples after PUI treatment at s2 and s3 irrespective of the instrumentation technique, 42% of the root canals after GWS + MIT and 50% after GWS + MIT had undetectable LPS. A previous study reported the average depth penetration for NaOCl with GWS is 447 µm, 4 × more than with PUI (112 µm)^37^. Given such depth penetration for NaOCl with GWS and the mean LPS depth penetration achieved with the current LPS dentin infection model (400 µm), it is not unreasonable to expect root canals with undetectable LPS. It is important to point out that the KQCL test used in this study for LPS quantification is the most sensitive LAL assay among the commercially available LAL tests, which can detect low LPS levels, as low as 0.005 EU/mL.

Despite the lack of studies evaluating the effectiveness of GWS in LPS disinfection, the higher effectiveness of GWS over PUI found here is consistent with previous disinfection studies^17,19^. Choi et al. comparing GWS to PUI, found that GWS showed a greater reduction in biofilm within the mesial roots of maxillary, mandibular molars, and mesiobuccal roots of maxillary molars^17^. Zhang et al. demonstrated a highly effective reduction of intracanal bacterial DNA with the GWS with better predictability than the ultrasonic system^19^. Moreover, previous studies have shown the enhanced tissue dissolution rate and cleaning ability of GWS^16,17,23^.

Although in most in vitro studies, it is possible to standardize the experimental groups to minimize confounding variables, GWS can offer limitations inherent to the technology and mechanism of action. However, we standardized the experimental groups by using 3% NaOCl in all groups tested in this study. In addition, we included a GWS group with conventional instrumentation (CIT) enlargement to ISO file size #35 (GWS + CIT) and a PUI group with minimally invasive instrumentation technique (MIT) with root canal enlargement to an ISO file size #15. It is worth pointing out that the high flow rate of the irrigant delivered with the GWS is not achievable with conventional irrigation using a syringe; therefore, it was not impossible to standardize it across the different groups tested.

In conclusion, GWS was the most effective protocol against LPS in infected root canals using MIT and CIT techniques.

## Material and methods

The local institutional review board at the University of Maryland, Baltimore, MD approved the collection of human extracted teeth used in this study (HP-00093483). All methods were performed following the review board at the University of Maryland guidelines and regulations. The need for obtaining informed consent was waived by the University of Maryland Baltimore ethics committee/IRB. A priori power analysis was performed using GPower (Version 3.1.9.2, Universität Kiel, Germany) to ensure that the results attained adequate power. The sample size calculation was based on a previous study^11^, which indicated 10 teeth per group. We included 12 teeth per group, considering the possibility of tooth loss through the study. A total of sixty freshly extracted human maxillary first premolars with two roots were obtained from the oral surgery department at the University of Maryland and included in this study. The inclusion criteria were as follows: teeth with one buccal and one palatal canal, no previous endodontic treatment, no calcifications, no internal resorption, and completely formed roots. The external root surfaces were cleaned with periodontal curettes. Teeth previously root canal treated, with extensive caries or restorations, crowns, cracks, or fractures were excluded. All teeth were sterilized using steam autoclave and kept hydrated in distilled water.

After access cavity preparation, the canals were explored with a K-file size #15 under the digital operator microscope, and patency obtained. We excluded teeth with root canal with the initial apical diameter larger than a K-file size #15. Twelve additional teeth with no LPS inoculation and no treatment were submitted to gamut radiation with cobalt 60 (20 KGy for 6 h) to degrade preexisting LPS^11,31^ and then cryogenically ground/ pulverized for quantification of LPS. Of the 60 teeth included, twelve were pretreated to open dentinal tubules, inoculated with fluorescent LPS conjugate (Alexa Fluor 594), and submitted to confocal laser scanning microscopy (CLSM) to verify the LPS penetration into the dentin and validate this novel LPS-infection model. Forty-eight teeth were randomly divided into groups according to treatment: PUI + MIT, PUI + CIT, GWS + MIT, GWS + CIT (all, n = 12). A person who was not involved in this study generated a random allocation sequence using computer software. Randomization codes were placed in opaque and sealed envelopes. The respective envelope was opened before the treatment.

### Dentin pre-treatment and LPS inoculation

Before dentin pretreatment, all teeth were accessed through the crown with a round bur #4. The canal was explored with a K-file size #10 to obtain patency under the digital operator microscope. Once the patency was confirmed, the working length (WL) was established to 1 mm short of the apical foramen. For the dentin pretreatment, first, all teeth were submitted to an ultrasonic bath for 10 min in 17% EDTA (Ethylenediaminetetraacetic acid), followed by 10 min in 5.25% NaOCl^38^. Second, root canals were soaked with 17% EDTA, and the tooth was placed in a 1.5 mL Eppendorf microtube (Corning, NY) and centrifuged at 1400×*g*, 2000×*g*, 3600×*g*, and 5600×*g* in sequence for 5 min each. The solution was renewed inside the root canal after each cycle. This protocol was repeated twice. The root canals were flushed with 10 mL of 5.25% NaOCl. After the tooth was placed back in the 1.5 mL plastic tube, the canals were soaked with 5.25% NaOCl and submitted to the centrifugation cycles listed above. Lastly, the NaOCl was inactivated with 5 mL sterile 0.5% sodium thiosulfate, with a final flush of 5 mL of endotoxin-free water (LAL water, LONZA, Walkersville). The canals were then dried with sterile/apyrogenic paper points.

For the LPS inoculation, we adopted a valid and commonly used bacterial dentin infection protocol described elsewhere^39^. Teeth were placed in a 1.5 mL plastic tube and inoculated with lipopolysaccharides from *Escherichia coli* Serotype 055:B5, Alexa Fluor 594 Conjugate solution (Thermo Fisher Scientific, Waltham, MA). The LPS concentration inoculated in the canals (10EU/mL) was according to Cardoso et al. study, which indicated a similar amount of LPS detected in primary root canal infections^40^. After, teeth were centrifuged at 1400×*g*, 2000×*g*, 3600×*g*, and 5600×*g* in sequence for 5 min each. This protocol was repeated twice.

After LPS inoculation, teeth were randomly assigned and allocated into the experimental groups listed above.

### Root canal procedures and LPS sampling

The root canals were sampled with paper points as described elsewhere^11^. The canals were irrigated with 1 mL of sterile/apyrogenic water, and the first LPS sample (s1) was collected. For s1, a sterile/apyrogenic paper point size #15 (Dentsply Sirona, Ballaigues, Switzerland) was introduced into WL in the buccal and palatal canals and left in position for 60 s. The LPS samples were placed in 1.5 mL sterile/apyrogenic tubes containing 1 mL of endotoxin-free water (LAL water, LONZA, Walkersville, MD, US) to quantify LPS further. After s1, the canals were instrumented according to the groups tested.

Before instrumentation, a #10 K-file was inserted in the canal, and patency was checked. The canals were instrumented with Vortex Blue rotary files (Dentsply, Sirona) at 500 rpm. For the minimally invasive instrumentation technique (MIT), the canals were enlarged to a size 15/0.04 with Vortex Blue rotary file at the WL. For the conventional instrumentation technique (CIT), the canals were enlarged to a size 35/0.04 with Vortex Blue rotary file at the WL. The canals were irrigated with 5 mL of 3% NaOCl after the use of each file. Patency was checked with a # 10 hand K-file between the files.

### Passive ultrasonic irrigation

For PUI groups (PUI + MIT and PUI + CIT), the tooth was irrigated using a 30-G side vented Maxi-i-Probe (DENTSPLY Maillefer, Tulsa Oklahoma) needle with 5 mL of 3% NaOCl. The NaOCl was activated SATELEC Sonofile (ACTEON Inc.) K-file ultrasonic tip size 15 for 1 min in each canal at a power setting of 4 using a ProUltra Piezo Ultrasonic unit (ProUltra, Dentsply Tulsa Dental). The ultrasonic file was taken 2 mm short of the root canal length. The canals were irrigated with 5 mL of 3% NaOCl. After, the canals were rinsed with 5 mL of 17% EDTA for 1 min and then rinsed off with 5 mL 3% NaOCl. This irrigation procedure was repeated twice for a total of 3 min 17% EDTA action. The canals were rinsed off with 5 mL of 3% NaOCl and inactivated with 5 mL of sterile 0.5% sodium thiosulfate for 1 min. Lastly, the canals were flushed with 5 mL of endotoxin-free water (LAL water, LONZA, Walkersville). After the PUI protocol, a second LPS sample (s2) was collected from the root canals as previously described.

### GentleWave system (GWS + MIT; GWS + CIT)

**Figure 1:**
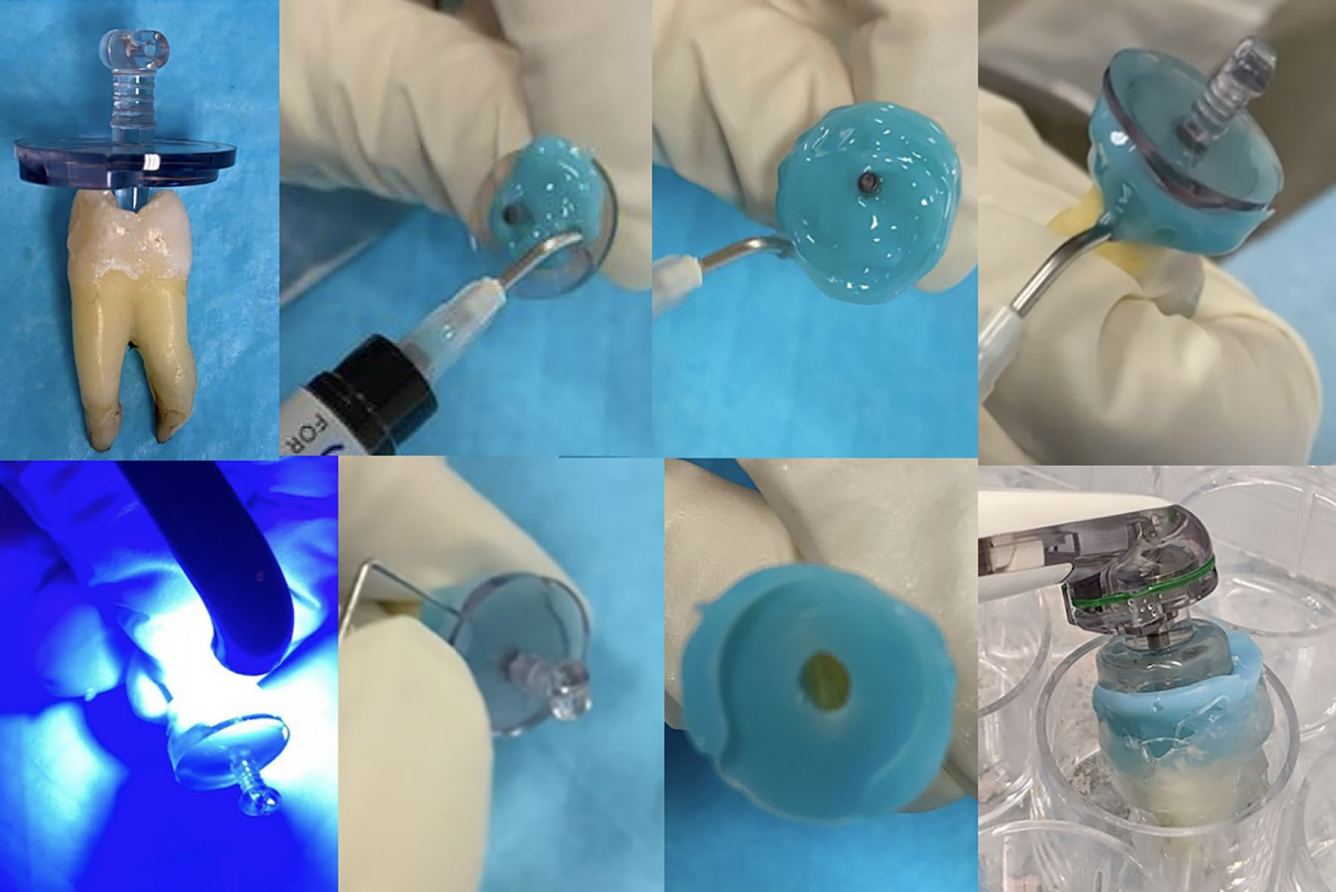
Occlusal platform building for the GentleWave System (GWS).

For GWS groups, a #10 hand K-file was placed inside the canal, and patency was checked. The GWS was used according to the manufacturer's instructions. First, we built an occlusal platform with a resin material (SoundSeal, Sonendo Inc.), following the manufacturer's instructions (Fig. 1). The teeth sample was stabilized in a 24-well pyrogen-free plate (Corning Costar, Cambridge, MA, USA). The GWS depth gauges were used to determine the proper sealing cap for the GWS handpiece. After building the GWS platform, the root and the apex were sealed with hot glue. The GWS was used with 3% NaOCl for 5 min, distilled water for 15 s, 17% EDTA for 2 min, and distilled water for 15 s as recommended by the manufacturer. After GWS cycle completion, NaOCl was inactivated with 5 mL of sterile 0.5% sodium thiosulfate for 1 min and rinsed with 5 mL of endotoxin-free water (LAL water, LONZA, Walkersville). Lastly, after the root canal procedure was completed, a second LPS sample (s2) was collected from the root canals as previously described.

### Cryogenic grinding technique for intraradicular LPS analysis

For intraradicular LPS analysis (s3), we innovatively cryogenically ground and pulverized the teeth. A similar grinding protocol was previously explored for bacterial analysis^1,30^. Teeth from all four experimental groups were cryogenically ground/pulverized for intraradicular LPS analysis. Twelve additional teeth with no LPS inoculation and no treatment were submitted to gamut radiation with cobalt 60 (20 KGy for 6 h) to degrade preexisting LPS^11,31^ then cryogenically ground/ pulverized for quantification of LPS.

Before grinding, all teeth were submitted to external disinfection, according to Siqueira et al.^30^. The external surfaces of the teeth were sampled with a sterile/ apyrogenic swab and stored at – 20 °C for future LPS analysis. Briefly, each tooth was placed into a cylindrical stainless tube and placed inside a 6770 SPEX SamplePrep Freezer/ Mill (Spex, Metuchen, NJ). The vial containing teeth was immersed in liquid nitrogen throughout the grinding cycle. This machine cools samples to cryogenic temperatures and pulverizes them by magnetically shuttling a steel impactor back and forth against two stationary end plugs. The cryogenic grinding included the following protocol: (a) precooling for 10 min, (b) first cycle, 1 min at a rate of 10 cycles per sec, (c) cool time, 1 min while the sample cools, (d) the second cycle, the sample is ground for 1 min, (e) cool time, 1 min while the sample cools, and (f) the third cycle, the sample is ground for 1 min. The total time for grinding was 15 min. After grinding, the tooth powder was transferred into a 15 mL centrifuge tube, and the third LPS sample (s3) was obtained for LPS quantification.

### Confocal laser scanning microscope (CLSM)

**Figure 2:**
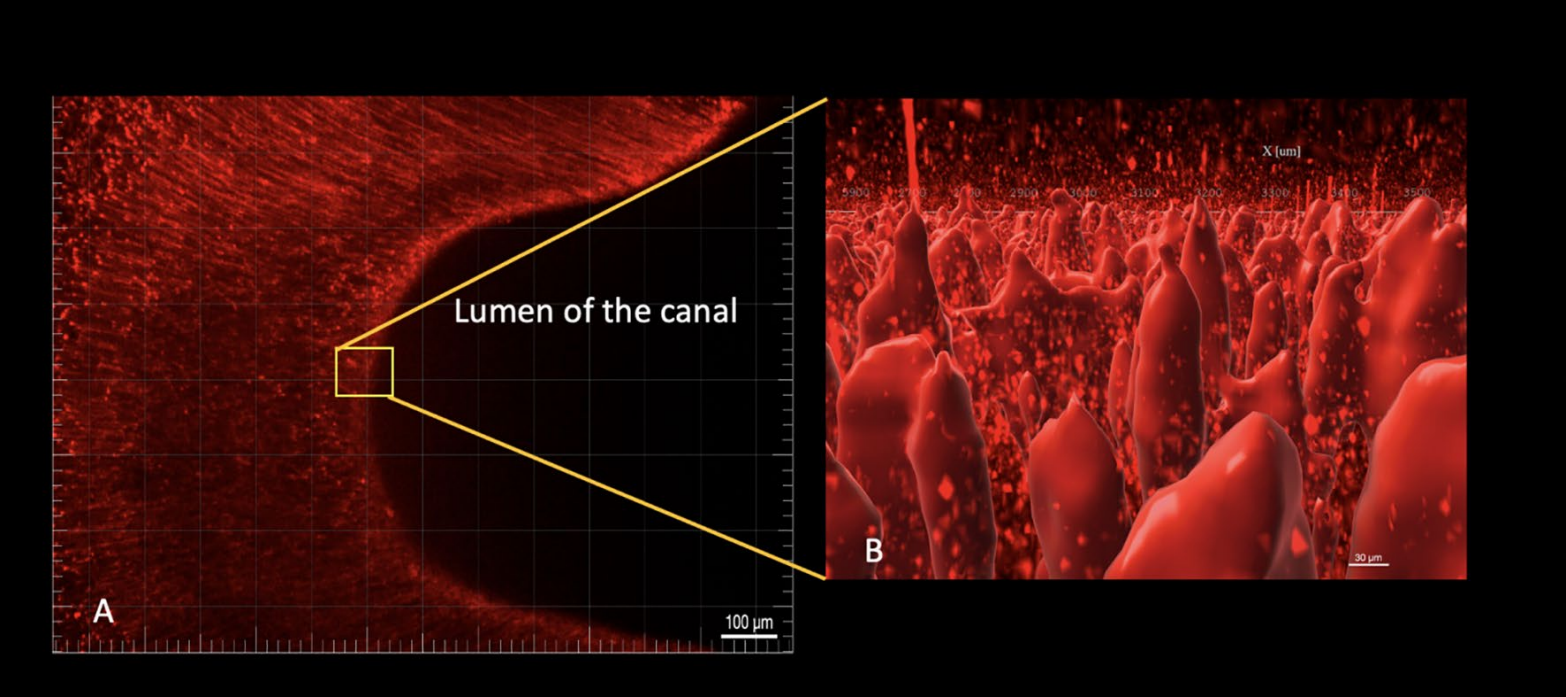
Fluorescent LPS-dentin infection model illustration: (**A**) Fluorescent LPS, cervical third of the root, Z, 10 ×; (**B**) 3D-reconstruction (Fluorescent LPS in the cervical third of the root, Z, 10 ×).

Of the 60 teeth included, twelve were dentin pretreated, inoculated with fluorescent LPS conjugate (Alexa Fluor 594), and submitted to confocal laser scanning microscopy (CLSM) to validate this novel LPS-infection model. Teeth were sectioned using 0.3 mm Isomet disc (Buehler, IL, USA) under constant cooling using distilled water. The roots were fixed on the Isomet with a low fusion impression compound (Kerr, MI, USA). Two 1 mm sections were taken from each root at the coronal, middle, and apical thirds. Teeth were analyzed using confocal laser scanning microscopy (Nikon W-1 Spinning Disk, Nikon Instruments Inc., Melville, NY) with the wavelength of absorption and fluorescence emission of 590/617 for visualization of Alexa Fluor 594 Alexa Fluor 594 conjugate. All samples were observed using the 5x, 10x, and 40 × oil lenses. The image samples were acquired 10 μm below the tooth surfaces. The images were analyzed using Imaris (Version 8.3.1; Bitplane) to verify the average of LPS linear penetration. Figure 2 illustrates this fluorescent LPS-dentin infection model.

### Quantification of LPS (KQCL test)

The quantification of LPS was performed using the Limulus Amebocyte Lysate (LAL) according to Martinho et al.^41^. The chromogenic kinetic test used to quantify LPS was the KQCL test (LONZA, Walkersville, MD). All samples (s1-s3) were reconstituted with 1 mL of LAL water (LAL water, LONZA). The KQCL test was performed following the manufacturers' instructions. First, we created a 96-well template in the WinKQCL software. The assay type was selected as Kinetic-QCL with the following default template parameters (Delta t = 150 s; Measurement filter = 405 nm; Delta mOD = 200; Number of reads = 40). Initially, 100 μL of the LAL reagent water blank, LPS standards (0.005, 0.05, 0.5, 5.0, 50 EU/mL), samples, and samples + spike were inoculated to a 96-well microplate following a template. After, the plate was pre-incubated for 10 min at 37 °C. Near the end of the pre-incubation period, the Kinetic-QCL reagent vials were reconstituted with 2.6 mL LAL reagent water and mixed gently. Next, 100 μL of the Kinetic-QCL reagent was added to all the microplate wells in the first column, continuing in sequence until the last column (A1–H1). The reagent was added quickly as recommended in the kit instructions, and the WinKQCL software initiated the test. Replicate samples were run to establish a good technique and low coefficient of variation. To verify possible product inhibition from each testing sample with the LAL reaction, samples were spiked with 10 μL of the 5.0 EU/ml solution into each well according to the manufacturer's instructions. The amount of LPS in the sample is calculated based on a polynomial curve-fitting model generated by the WinKQCL software.

Data obtained from the quantification of LPS were tabulated on a spreadsheet and analyzed using Graph-Pad Prism software, version 6.01 (GraphPad Software, San Diego, CA, USA). The Shapiro–Wilk test was used to assess the normal distribution of the data. The paired t-test was used for intragroup analysis, and one-way ANOVA for intergroup analysis. All statistical tests were performed at a significance of 5% (P < 0.05).
